# Homer2 deletion alters dendritic spine morphology but not alcohol-associated adaptations in GluN2B-containing N-methyl-D-aspartate receptors in the nucleus accumbens

**DOI:** 10.3389/fphar.2015.00028

**Published:** 2015-02-23

**Authors:** Natalie S. McGuier, Audrey E. Padula, Patrick J. Mulholland, L. Judson Chandler

**Affiliations:** Department of Neuroscience, Charleston Alcohol Research Center, Medical University of South Carolina, Charleston, SC, USA

**Keywords:** dendritic spines, nucleus accumbens, structural plasticity, cellular morphology, Homer2, postsynaptic density

## Abstract

Repeated exposure to ethanol followed by withdrawal leads to alterations in glutamatergic signaling and impaired synaptic plasticity in the nucleus accumbens (NAc) in both clinical and preclinical models of ethanol exposure. Homer2 is a member of a family of postsynaptic density (PSD) scaffolding proteins that functions in part to cluster N-methyl-D-aspartate (NMDA) signaling complexes in the PSD, and has been shown to be critically important for plasticity in multiple models of drug and alcohol abuse. Here we used *Homer2* knockout (KO) mice and a chronic intermittent intraperitoneal (IP) ethanol injection model to investigate a potential role for the protein in ethanol-induced adaptations in dendritic spine morphology and PSD protein expression. While deletion of *Homer2* was associated with increased density of long spines on medium spiny neurons of the NAc core of saline treated mice, ethanol exposure had no effect on dendritic spine morphology in either wild-type (WT) or Homer2 KO mice. Western blot analysis of tissue samples from the NAc enriched for PSD proteins revealed a main effect of ethanol treatment on the expression of GluN2B, but there was no effect of genotype or treatment on the expression other glutamate receptor subunits or PSD95. These data indicate that the global deletion of *Homer2* leads to aberrant regulation of dendritic spine morphology in the NAc core that is associated with an increased density of long, thin spines. Unexpectedly, intermittent IP ethanol did not affect spine morphology in either WT or KO mice. Together these data implicate Homer2 in the formation of long, thin spines and further supports its role in neuronal structure.

## INTRODUCTION

Chronic intermittent ethanol exposure and withdrawal induce plasticity in glutamatergic synapses of the nucleus accumbens (NAc). These neuroadaptations include altered synaptic expression of N-methyl-D-aspartate (NMDA)-type glutamate receptor subunits (Gremel and Cunningham, 2009; Obara et al., 2009; Mulholland et al., 2011), increased extracellular glutamate levels in ethanol-seeking behaviors (Gass et al., 2014; Griffin et al., 2014), and morphological adaptations in the size and density of dendritic spines in the NAc (Zhou et al., 2007; Spiga et al., 2014). Furthermore, these glutamate-based neuroadaptations are thought to contribute to the severity of withdrawal symptoms, consumption of large amounts of ethanol (EtOH), and relapse-seeking behaviors (Kalivas, 2009).

Dendritic spine morphology and NMDA receptor expression are intimately associated with the form and function of the postsynaptic density (PSD), an area of dense protein scaffolding in the postsynaptic membrane (Sheng and Sala, 2001; Kristiansen et al., 2006; Cui et al., 2007; Ultanir et al., 2007). The Homer family of proteins (Homer1a/b/c, 2a/b, and Homer3a/b) plays an integral role in the structure of the PSD by tethering metabotropic glutamate (mGlu) receptors, IP_3_ receptors, and ionotropic glutamate receptors to scaffolding proteins through an Enabled/VASP homology 1 (EVH1) domain (Xiao et al., 1998; Shiraishi-Yamaguchi and Furuichi, 2007). Homer-linked structures are thought to act as a signaling complex by linking various proteins in close proximity, ultimately facilitating signal transduction. For example, mGlu receptors, IP_3_ receptors, and Shank are all critically involved in Ca^2+^ signaling at the PSD, and are bound in proximity to each other by Homer and other scaffolding proteins. Subsequently, activity-dependent reversible clustering of these Homer complexes could lead to structural and functional remodeling of the synapse (Shiraishi-Yamaguchi and Furuichi, 2007; Shiraishi-Yamaguchi et al., 2009). Evidence also suggests that Homer2 regulates expression of the GluN2A subunit of the NMDA receptor in the accumbens (Szumlinski et al., 2005). Because Homer2a/b is highly expressed throughout the CNS and is predominant in the PFC, NAc, and striatum (Shiraishi-Yamaguchi and Furuichi, 2007), this member of the Homer family is of particular interest when studying neuronal plasticity at the synaptic level.

In models of cocaine addiction, Homer2 has been shown to regulate behavioral and biochemical sensitivity to the drug (Szumlinski et al., 2004). A plethora of recent study also implicate Homer2 in the maladaptive behavioral plasticity associated with ethanol reward and addiction (Szumlinski et al., 2005; Obara et al., 2009; Goulding et al., 2011; Cozzoli et al., 2012). Homer2a/b and mGlu1/5 expression are increased in the NAc after chronic intermittent intraperitoneal (IP) injections of ethanol (Szumlinski et al., 2005; Goulding et al., 2011). These studies suggest that ethanol exposure can produce relatively long-lasting adaptations of Homer2 expression and glutamatergic signaling in the NAc. In the present study, we investigated how the deletion of *Homer2* impacted dendritic spine morphology and protein expression in PSD-enriched tissue from the NAc using a chronic intermittent IP ethanol exposure model. These studies revealed that this chronic intermittent ethanol exposure paradigm resulted in increased GluN2B expression in both wild-type (WT) and knockout (KO) mice, and that *Homer2* deletion is associated with an increase in the density of long, thin spines. Taken together, these observations provide evidence that Homer2 plays a role in the regulation of dendritic spine morphology, and further suggests that homeostatic regulation of GluN2B in response to ethanol exposure is robust enough to overcome the absence of Homer2.

## MATERIALS AND METHODS

### CHRONIC INTERMITTENT IP ETHANOL EXPOSURE

Adult (8–10 weeks of age at the start of experimentation) male WT mice and mice with null mutations of *Homer2* (backcrossed with C57BL/6J mice for >6 generations) were generated and maintained by heterozygous mating as described previously (Szumlinski et al., 2005). Genotype for each mouse was determined in duplicate, and only mice with confirmed genotype were included in the study. Mice were group housed (3–4/cage) under a reverse 12 h light/dark cycle (lights on at 0200). Rodent chow (Harlan Teklad, Madison, WI, USA) and water were available *ad libitum*. Mice were maintained in an Association for Assessment and Accreditation of Laboratory Animal Care (AAALAC) International-accredited facility with automated temperature, humidity, and light cycle control. Following a protocol previously reported to induce locomotor sensitization and conditioned place preference (Nocjar et al., 1999; Szumlinski et al., 2005), mice received intraperitoneal (IP) injections of vehicle (0.9% sterile saline) or 3 g/kg ethanol (in 0.9% sterile saline; 0.2 ml/g) every other day for a total of eight injections. All work was approved by the Institutional Animal Care and Use Committee and conducted according to the requirements of the NIH Guide for the Care and Use of Laboratory Animals (2011).

### BLOOD ETHANOL CONCENTRATION

To determine genotypic differences in ethanol metabolism and any development of metabolic tolerance, blood samples were taken from the infraorbital sinus from WT and *Homer2* KO mice 1 h after the first and last ethanol injection. Blood ethanol concentrations (BECs; mg%) were analyzed using a modified version of a previously described colorimetric alcohol oxidase assay (Prencipe et al., 1987).

### ETHANOL-INDUCED SEDATION

To assess the effect of deletion of Homer2 on the sedative and motor-impairing effects of ethanol, we utilized an ethanol-sedation test using previously described methods (Szumlinski et al., 2005). Knock out and WT mice received intraperitoneal (IP) injections of 5 g/kg ethanol. Once immobile, the mice were laid on their backs in their home cages and the time to regain their righting reflex as defined by the time taken to place all four paws on the cage floor was measured.

### SUBCELLULAR FRACTIONATION AND WESTERN BLOTTING

Tissue punches were taken from the NAc core of WT and *Homer2* KO mice 1 h after the final ethanol or saline injection. Triton X-100 insoluble fractions that are enriched in PSD proteins were prepared as previously described (Mulholland et al., 2011). In brief, a Dounce homogenate was prepared and centrifuged at 12,000 × *g* for 20 min to obtain a membrane fraction. The pellet was resuspended in buffer containing 0.5% Triton X-100 and rotated at 4°C for 15 min. This fraction was then centrifuged at 12,000 × *g* for 20 min to yield soluble and insoluble fractions. The insoluble fraction was then sonicated in 2% LDS and stored at -80°C until analysis.

For western blot procedures samples were diluted with NuPAGE 4X LDS sample loading buffer (Invitrogen Corporation, Carlsbad, CA, USA; pH 8.5) containing 50 mM dithiothreitol, and samples were denatured for 10 min at 70°C. Five micrograms of each sample were separated using the Bis-Tris (375 mM resolving buffer and 125 mM stacking buffer, pH 6.4; 7.5% acrylamide) discontinuous buffer system with MOPS electrophoresis buffer (50 mM MOPS, 50 mM Tris, 0.1% SDS, 1 mM EDTA, pH 7.7). Protein was then transferred to Immobilon-P Polyvinylidene fluoride (PVDF) membranes (Millipore, Bedford, MA, USA) using a semi-dry transfer apparatus (Bio-Rad Laboratories, Hercules, CA, USA). After transfer, blots were washed with phosphate-buffered saline containing 0.1% Tween 20 (PBST) and then blocked with PBST containing 5% non-fat dried milk (NFDM) for 1 h at room temperature with agitation. The membranes were then incubated overnight at 4°C with primary antibodies diluted in PBST containing 0.5% NFDM and washed in PBST prior to 1 h incubation at room temperature with horseradish peroxidase conjugated secondary antibodies diluted 1:2000 in PBST. Membranes received a final wash in PBST and the antigen-antibody complex was detected by enhanced chemiluminescence using a ChemiDoc MP Imaging system (Bio-Rad Laboratories, Hercules, CA, USA). The bands were quantified by mean optical density using computer-assisted densitometry with ImageJ v1.41 (National Institutes of Health, USA). Because the use of loading controls [e.g., actin, Glyceraldehyde 3-phosphate dehydrogenase (GAPDH)] for normalization in western blot experiments are subject to quantitation errors (Dittmer and Dittmer, 2006; Aldridge et al., 2008), normalization to a total protein stain (i.e., amido black) was used in these studies. Before each study, a series of western blots were performed using different titrations of sample and antibody to establish the linear range for each target. GluN1 antibody was purchased from BD Pharmingen (1:4000; Catalog # 556308; San Jose, CA, USA). GluN2A and GluA1 antibodies were purchased from EMD Millipore (1:2000; Catalog # 07-732; Billerica, MA, USA). GluN2B and PSD95 antibodies were purchased from the UC Davis/NIH NeuroMab Facility (1:2000; Catalog # 75-097; Davis, CA, USA).

### DENDRITIC SPINE LABELING, MORPHOLOGICAL CLASSIFICATION, AND ANALYSIS

Neuronal labeling and morphological classification of dendritic spines of medium spiny neurons (MSNs) in the NAc core were carried out using previously reported methods (Jung et al., 2013; McGuier et al., 2014). One hour after the final ethanol injection, mice were anesthetized with urethane (1.5 g/kg, IP) and perfused with 0.1 M phosphate buffer (PB) followed by 1.5% paraformaldehyde (PFA) in PB. Brains were blocked and post-fixed for 30 min. Next, 150 μm thick coronal slices were prepared using a vibratome. DiI coated tungsten particles (1.3 μm diameter) were delivered to the slices using a modified Helios Gene Gun (Bio-Rad; Hercules, CA, USA) fitted with a polycarbonate filter (3.0 μm pore size; BD Biosciences; San Jose, CA, USA). Slices were left overnight at 4°C in PB to allow the DiI to completely diffuse through labeled neurons and sections were post fixed in 4% PFA for 1 h at room temperature. After mounting with Prolong Gold Antifade mounting media (Life Technologies; Carlsbad, CA, USA), slices were imaged (1–4 dendritic sections/mouse; voxel size: 47 nm × 47 nm × 100 nm) using a Zeiss LSM 510 confocal microscope fitted with a 63 × oil immersion objective (Plan-Apochromat, Zeiss, NA = 1.4, working distance = 190 μm). Images of sections of dendrites starting >75 μm away from the soma of MSNs in the NAc core were acquired and then deconvolved using AutoQuant (Media Cybernetics; Rockville, MD, USA). Imaris XT (Bitplane; Zurich, Switzerland) was used to generate a filament of the dendritic shaft and spines. Dendritic spines were identified using Imaris software and then classified into four categories (stubby, long, filopodia, and mushroom) based on the spine length and the width of the spine head and neck, where *L* is spine length, *D*_H_ is spine head diameter, and *D*_N_ is spine neck diameter. Long spines were identified as having a *L* ≥ 0.75 μm and <3 μm, mushroom spines had a *L* < 3.5 μm, *D*_H_ > 0.35 μm and a *D*_H_ > *D*_N_, stubby spines had a *L* < 0.75 μm, and filopodia were identified as having a *L* ≥ 3 μm. Data on dendritic spine parameters were averaged for each dendritic section and were collated from the Imaris output via custom scripts written in Python. Dendritic spine data were then averaged for each mouse, and the data were analyzed using two-way ANOVAs and unpaired, two-tailed *t*-tests. All data are reported as mean ± SEM and statistical significance was established with *p* < 0.05.

## RESULTS

### *Homer2* KO AND REPEATED ETHANOL EXPOSURE

**FIGURE 1:**
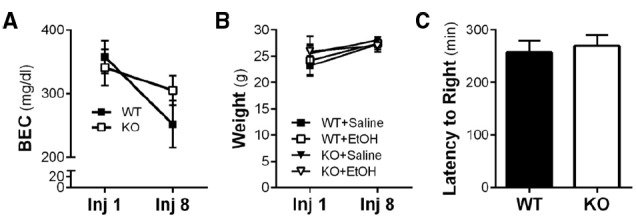
**Ethanol-induced behavior and metabolic adaptations are not altered by *Homer2* KO. (A)** Neither WT nor KO mice developed metabolic tolerance to ethanol after the final IP injection and there was no effect of genotype on ethanol metabolism. **(B)** Wild-type and KO mouse weights were not different from each other and did not change during the course of the chronic intermittent ethanol IP injection procedure. **(C)** There was no effect of genotype on loss of righting reflex in response to a single 5 g/kg IP injection of ethanol.

It has been reported that *Homer2* KO mice exhibit enhanced aversion to ethanol, fail to sensitize to repeated injections of ethanol, and display increased latency to regain a righting reflex (Szumlinski et al., 2005). In our initial set of studies, we sought to replicate these behavioral and metabolic experiments as a means of confirming the behavioral phenotype of *Homer2* deletion in our cohort of mice. After the first and last of eight IP injections of ethanol, the blood ethanol concentration (BEC) was determined in WT and KO mice. As shown in Figure 1A, there was no interaction of genotype and time in BECs of the mice after the eighth injection compared to the first injection [repeated-measures two-way ANOVA *F*(1,9) = 4.8; *p* = 0.0552; *n* = 4–7/group]. However, there was a main effect of time on BEC [F(1,9) = 19.70; *p* = 0.0016]. Also shown in Figure 1B, body weights did not differ by genotype or treatment when measured after the first and last injection. Lastly, in a latency to right task, WT and *Homer2* KO mice were administered a sedative dose of ethanol (5 g/kg IP) and the time until the mice regained its righting reflex was determined. In contrast to previously published work (Szumlinski et al., 2005), deletion of *Homer2* did not influence latency to right (Figure 1C; *n* = 7–8/genotype). Therefore, *Homer2* KO mice did not exhibit changes in ethanol metabolism or body weight after repeated ethanol exposure or latency to right after a challenge injection of acute ethanol.

### REPEATED ETHANOL EXPOSURE, *Homer2*, AND PSD PROTEINS

**FIGURE 2:**
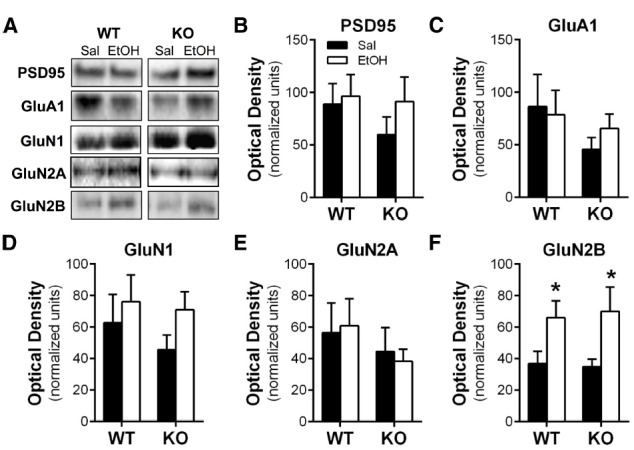
**Repeated chronic intermittent injections of ethanol induced NMDA receptor plasticity independent of genotype. (A)** Representative images of western blots of the PSD-enriched tissue samples. There was no effect of ethanol treatment or genotype on the expression of **(B)** PSD-95, **(C)** GluA1, **(D)** GluN1, or **(F)** GluN2A. **(E)** Repeated intermittent injections of ethanol resulted in increased expression of GluN2B in both WT and *Homer2* KO mice (*p < 0.05).

Homer2 plays an integral role in the scaffolding network of the PSD, and repeated bouts of ethanol exposure and withdrawal are known to induce adaptations in the expression of glutamate receptors at synapses in the NAc (Zhou et al., 2007; Spiga et al., 2014; Uys et al., in press). Thus, we hypothesized that the absence of the Homer2 protein would prevent these changes in ethanol-induced neuroplasticity. To test this hypothesis, WT and KO mice were subjected to the chronic intermittent IP ethanol injection procedure, and a PSD-enriched faction was prepared 1 h after the last injection. As shown in Figure 2, western blot analysis revealed that there was no significant treatment effect or interaction of genotype on the expression levels of PSD95, GluN1, GluN2A, or GluA1, but there was a significant effect of ethanol on the expression of GluN2B in both WT and KO mice [main effect of treatment *F*(1,27) = 8.315, *p* = 0.0076]. These data indicate that while chronic intermittent IP injections of ethanol induces biochemical adaptations in GluN2B expression, this increase does not depend on Homer2 expression.

### *Homer2* AND DENDRITIC SPINES

**FIGURE 3:**
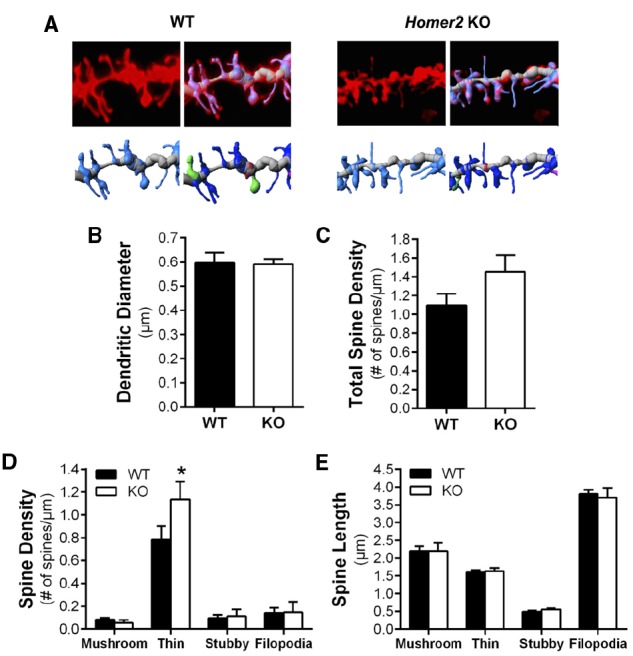
**Deletion of Homer2 increased long spine density compared to WT mice. (A)** Representative images of diolistically labeled dendritic segments from WT saline and Homer2 KO saline treated mice. The dendritic shaft is shown in gray and spines are shown in light blue. Classified spines are b color (dark blue = long, thin; pink = filopodia; red = stubby; green = mushroom). **(B)** Dendritic diameter and **(C)** total spine density were not altered by genotype. **(D)** Analysis of spine subclasses revealed that long thin spine density is increased in KO mice compared WT (*p < 0.05). **(E)** There were no genotypic differences in spine length.

Repeated cycles of ethanol exposure and withdrawal are known to induce adaptations in the morphology of dendritic spines in MSNs of the NAc core and shell (Zhou et al., 2007; Spiga et al., 2014; Uys et al., in press). Given that Homer2 is a key component of the PSD scaffold, we next investigated whether the genetic deletion of this protein altered structural plasticity by examining spine morphology in the MSNs of the NAc core. Analyses comparing saline-injected WT and KO mice revealed no differences in total spine density [two-tailed *t*-test, *t*(15) = 1.682, *p* = 0.1133, *n* = 6–11 mice/genotype, 11–23 dendritic segments/genotype; Figures 3A,C], or the diameter of the dendritic shaft [two-tailed *t*-test, *t*(15) = 0.1011, 0.9208; Figure 3B]. Dendritic spines can be classified into subclasses (stubby, mushroom, long, or filopodia) based on the morphological characteristics of the spine. Length and diameter of the spine neck and terminal point, and the shape of the spine have been associated with differential expression of glutamate receptors and Ca^2+^- and cAMP-regulated signaling proteins (Zhou et al., 2007; Obara et al., 2009; Goulding et al., 2011; Cozzoli et al., 2012; Lum et al., 2014). Comparison of these spine parameters showed no difference in spine length [two-way ANOVA, *F*(3,52) = 0.1606, *p* = 0.9224, *n* = 6–11 mice/genotype, 11–23 dendritic segments/genotype; Figure 3E], while the density of long, thin spines was significantly increased in the KO mice compared to WT [two-way ANOVA, *F*(3,60) = 2.551, *p* = 0.0640; *post hoc p* < 0.05; *n* = 6–11/genotype, 11–23 dendritic segments/genotype; Figure 3D].

**FIGURE 4:**
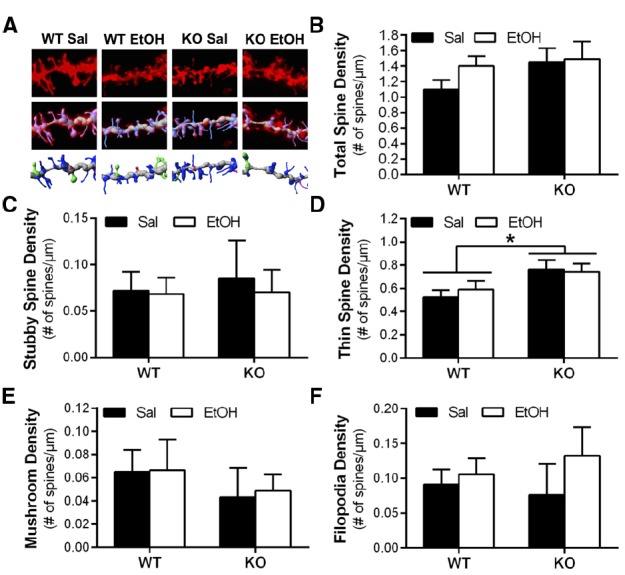
**Deletion of Homer2 is associated with increased long spine density compared to WT mice that is unaffected by repeated ethanol exposure. (A)** Representative images of WT and Homer2 KO saline (sal) and ethanol (EtOH) treated mice showing a diolistically labeled dendrite segment (top), a labeled dendrite with a computer-generated filament (middle), and the filament with classified spines (blue = long thin, pink = filopodia, red = stubby, green = mushroom; bottom). **(B)** Total spine density was unaffected by genotype or treatment. **(C)** Class analyses of spine density showed no effect of treatment or genotype on stubby spine density. **(D)** There was a main effect of genotype on thin spine density (*p = 0.0122), but no effect of treatment. Similar analyses showed no effect of treatment or genotype on the density of **(E)** mushroom spines or **(F)** filopodia. Representative images for WT Sal and KO Sal are the same as those used in Figure 3.

Wild-type and *Homer2* KO mice also received chronic intermittent IP ethanol injections to determine if Homer2 regulates ethanol-induced adaptations in dendritic spine morphology. In contrast to our hypothesis, chronic intermittent IP injections of ethanol had no effect on dendritic spine morphology or density, and there was no interaction between genotype and ethanol treatment on total spine density [two-way ANOVA, *F*(1,27) = 0.6362, *p* = 0.4320, *n* = 6–11 mice/group, 11–23 dendritic segments/group] or spine density by class [two-way ANOVAs, thin: *F*(1,27) = 0.3708, *p* = 0.5477; mushroom: *F*(1,27) = 0.008345, *p* = 0.9279; stubby: *F*(1,27) = 0.04343, *p* = 0.8365; filopodia: *F*(1,27) = 0.4314, *p* = 0.5169; *n* = 6–11 mice/group, 11–23 dendritic segments/group; Figures 4A–F]. In the case of long spine density, deletion of Homer2 lead to increased density regardless of treatment [two-way ANOVA, main effect of genotype *F*(1,27) = 7.221, *p* = 0.0122; *n* = 6–12 mice/treatment; Figure 4C]. Together these data indicate that Homer2 deletion increases density of long, thin spines.

## DISCUSSION

This study investigated the involvement of the PSD scaffolding protein Homer2 in ethanol-induced biochemical and morphological adaptations of the NAc using a chronic intermittent IP ethanol injection model. These studies showed no effect of intermittent IP ethanol exposure or genotype on ethanol metabolism, weight, or latency to right when comparing WT and *Homer2* KO mice. The expression of GluN2B was elevated in the PSD from both WT and KO mice after repeated ethanol exposure, however there was no influence of genotype on protein expression. Finally, while *Homer2* KO mice exhibited an increase in the density of long, thin spines in the NAc core compared to WT mice, chronic intermittent IP injections of ethanol did not induce morphological adaptations in this brain region.

It has been suggested that the Homer family of proteins are potential key regulators of dendritic spine morphology (Sala et al., 2002, 2005). In cultured hippocampal neurons, Sala et al. (2002) showed that dendritic spines on immature neurons overexpressing Shank were shorter and displayed thicker heads that morphologically resembled more mature type spines. Furthermore this study showed that a Homer1b-Shank interaction was necessary for this phenomenon. Additional studies in HeLa cells have shown that Cupidin’s (a Homer2a isoform) interaction with the small GTPase Cdc42 within a scaffolding network is required to prevent Cdc42-induced filopodia-like protrusion formation (Shiraishi-Yamaguchi et al., 2009). This group also showed that without Cupidin localized to the synapse in cultured hippocampal neurons, there was a marked decrease in miniature excitatory postsynaptic currents. Altogether these studies suggest that, at least in culture, Homer is necessary for spine maturation and synaptic function. In the present study, we observed that Homer2 deletion leads to an increase in the density of long spine on MSNs of the NAc core under basal conditions. This class of spine is characterized by its long, thin appearance, no significant head and neck separation, and a lack of a substantial PSD network (Jones and Powell, 1969; Peters and Kaiserman-Abramof, 1970; Uys et al., in press). Interestingly, long spines are associated with enhanced plasticity, and are believed to be precursors to the more stable and mature mushroom type “memory” spines (Holtmaat et al., 2006; Bourne and Harris, 2007; Arnsten et al., 2012), and recently it was reported that chronic ethanol exposure altered spine density in the NAc (Zhou et al., 2007; Spiga et al., 2014; Uys et al., in press). While we observed that Homer2 deletion did not alter the expression of PSD95 in PSD-enriched samples from the NAc, it is possible that the absence of Homer2 destabilizes PSD formation and promotes the formation of immature long spines. Indeed previous reports have shown a necessity of a Homer/c interaction to induce spine head enlargement via recruitment of IP_3_ receptors and subsequently ER cisternas in cultured hippocampal neurons (Sala et al., 2002, 2005).

In contrast with the influence of genotype on spine morphology, we did not observe an effect of chronic intermittent IP injection of ethanol on spine morphology or an interaction between genotype and ethanol treatment on long spine density. Although speculative, the intermittent IP ethanol exposure paradigm used in the present study could explain this lack of effect. Several rodent models of ethanol exposure have been shown to induce morphological adaptations in dendrites and dendritic spines in the NAc. In a free-choice drinking model, alcohol-preferring rats given either continuous access or repeated deprivation of ethanol resulted in fewer spines with larger heads in MSNs of the NAc (Zhou et al., 2007). In a separate study, alcohol-dependent rats on a liquid diet exhibited decreased overall spine density in the NAc shell, with long spines being selectively decreased (Spiga et al., 2014). In addition, we have previously shown that chronic intermittent ethanol exposure by vapor inhalation resulted in an overall increase in dendritic spine density in the NAc core that was attributed to an increase in long spine density (Uys et al., in press). Unfortunately, we were unable to test the *Homer2* KO mice in this dependence model because our preliminary experiments showed reduced survival rates during the withdrawal phase (unpublished observation), suggesting that *Homer2* KO mice are more sensitive to withdrawal-induced hyperexcitability than WT mice. Though the intermittent IP ethanol exposure paradigm used in the current study has been previously reported to induce biochemical adaptations in mice (Szumlinski et al., 2005; Goulding et al., 2011), in our hands this model did not result in changes in dendritic spine morphological plasticity in the NAc core. However, we did observe that *Homer2* KO mice have alterations in the density of long spines similar to the morphological phenotype in this region reported in other models of chronic ethanol exposure. As other abused substances (e.g., cocaine, heroin, and methamphetamine) produce morphological adaptations in dendritic spines, future studies should determine a role for Homer2 in regulation of spine density and morphology in other models of chronic drug exposure.

Increased expression of NMDA receptor subunits is associated with repeated ethanol exposure (Carpenter-Hyland and Chandler, 2007; Nagy, 2008). In particular, there is a marked increase in the expression and function of NMDA receptors in the NAc in continuous access and repeated withdrawal models of ethanol exposure (Zhou et al., 2007; Spiga et al., 2014). It is thought that this may represent a compensatory adaptation to counterbalance the inhibitory effect on neuronal excitability (Carpenter-Hyland and Chandler, 2006). Using western blot analysis we examined the expression of GluA1, NMDA receptor subunits and the scaffolding protein PSD95 1 h after the final injection. Consistent with previous findings, WT ethanol-exposed mice exhibited increased expression of the GluN2B subunit compared to saline controls, validating the ability of the intermittent IP injection paradigm of exposure to induce biochemical adaptations in NMDA receptors. We also observed that *Homer2* KO mice exhibited the same ethanol-induced increase in GluN2B expression. This finding is particularly interesting given that Homer2 clusters with NMDA receptors containing the GluN2B subunit (Shiraishi-Yamaguchi and Furuichi, 2007). These data therefore suggest that while Homer2 may play a role in PSD plasticity, increased GluN2B expression by ethanol is mediated through an additional process that is independent of Homer2.

In contrast to previous work showing a reduction in GluN2A expression in the accumbens of Homer2 KO mice (Szumlinski et al., 2005), we did not observe an effect of genotype on the expression of GluN2A. This discrepancy may be explained by the fractionation method used in preparation of the western blot samples. In the present study, a synaptosomal fraction was obtained and treated with Triton X-100 to isolate PSD-enriched proteins, yet in the previous studies western blot analysis was carried out using a total synaptosomal fraction (i.e., one containing proteins from both the synaptic and extrasynaptic membrane; Toda et al., 2003). Our data may therefore indicate that the KO-induced decrease in GluN2A might be restricted to the extrasynaptic pool of receptors.

Our studies attempted to link ethanol-induced behavior and *Homer2* KO by measuring latency to right in both WT and KO mice to confirm that the previously reported behavioral phenotypes were maintained in the current cohorts of mice (Szumlinski et al., 2005). Unexpectedly, we did not observe an effect of genotype on righting behavior after a sedating dose of acute ethanol. This is in direct contrast to previously published work (Szumlinski et al., 2005), which showed that the *Homer2* KO mice are more sensitive to the sedative effects of ethanol compared to their WT littermates. One potential explanation for this discrepancy could be genetic drift, which describes the tendency of genes to continuously evolve without selective pressure (Silver, 1995). As an example of this phenomenon, extensive homozygotic inbreeding of 5-HT_1B_ KO mice resulted in the loss of a unique drinking phenotype (Bouwknecht et al., 2000). To avoid genetic drift, mutant and control (C57BL/6J) populations should occasionally be interbred and the resulting heterozygotic offspring should be used to breed KO animals (Phillips et al., 1999). We implemented this breeding schema, suggesting that it is unlikely that genetic drift can account for the lack of effect of genotype on ethanol sedation in the present study. It is also possible that the progenitor strain (129Xi/SvJ; Shin et al., 2003), from which the embryonic stem cells (ES) were derived to generate the *Homer2* KOs, is a phenotypic match for the predicted ethanol-induced behavioral adaptations associated with *Homer2* deletion. This was the case in a dopamine receptor 2 deficient mouse where the ES progenitor strain (129/SvEv) used to generate the mutant mouse strain endogenously exhibited the same locomotor deficits as was predicted for the mutant. As a result, the mutant mouse performed the same as the inbred 129/SvEv mouse strain despite backcrossing the mutant with C57BL/6 mice (Kelly et al., 1998). In the case of the *Homer2* KO mice, it is possible that the effect of ethanol on loss of righting originally seen by Szumlinski et al. (2005) might have been a phenotype endogenous to 129Xi/SvJ mice that dissipated in our colony as the *Homer2* KO mice were continually crossbred with C57BL/6J mice over several more generations.

In summary, the results of the present study show that while chronic intermittent IP injections of ethanol induce increases in GluN2B expression in NAc of both WT and *Homer2* KO mice, deletion of *Homer2* does not effect this ethanol-induced adaptation. A lack of effect of intermittent IP ethanol exposure on dendritic spine morphology in both WT and KO mice indicate that the IP ethanol injection model of exposure does not appear to be sufficient to induce morphological adaptations. Finally, these studies show that deletion of *Homer2* leads to an increase in long thin spines of MSNs in the NAc core, providing the first evidence that *Homer2* directly affects dendritic spine morphology *in vivo*.

### Conflict of Interest Statement

The authors declare that the research was conducted in the absence of any commercial or financial relationships that could be construed as a potential conflict of interest.
